# Clinical Characteristics and Long-term Follow-up of Patients with Diabetes Due To *PTF1A* Enhancer Mutations

**DOI:** 10.1210/clinem/dgaa613

**Published:** 2020-09-07

**Authors:** Huseyin Demirbilek, Atilla Cayir, Sarah E Flanagan, Ruken Yıldırım, Yılmaz Kor, Fatih Gurbuz, Belma Haliloğlu, Melek Yıldız, Rıza Taner Baran, Emine Demet Akbas, Meliha Demiral, Edip Ünal, Gulcin Arslan, Dogus Vuralli, Gonul Buyukyilmaz, Sara Al-Khawaga, Amira Saeed, Maryam Al Maadheed, Amel Khalifa, Hasan Onal, Bilgin Yuksel, Mehmet Nuri Ozbek, Abdullah Bereket, Andrew T Hattersley, Khalid Hussain, Elisa De Franco

**Affiliations:** 1 Hacettepe University Faculty of Medicine, Department of Pediatric Endocrinology, Ankara, Turkey; 2 Diyarbakır Children’s Hospital, Clinics of Pediatric Endocrinology, Diyarbakir, Turkey; 3 Erzurum Training and Research Hospital, Clinics of Pediatric Endocrinology, Erzurum, Turkey; 4 Institute of Biomedical and Clinical Science, University of Exeter Medical School, Exeter, UK; 5 Adana Training and Research Hospital, Clinics of Pediatric Endocrinology, Adana, Turkey; 6 Cukurova University Faculty of Medicine, Department of Pediatric Endocrinology, Adana, Turkey; 7 Yeditepe University School of Medicine, Department of Pediatric Endocrinology, Istanbul, Turkey; 8 Kanuni Sultan Suleyman Training and Research Hospital, Clinics of Pediatric Endocrinology, Istanbul, Turkey; 9 Istanbul University, Istanbul Faculty of Medicine, Department of Pediatric Endocrinology, Istanbul, Turkey; 10 Gazi Yasargil Training and Research Hospital, Pediatric Endocrinology, Diyarbakır, Turkey; 11 University of Health Science, Behcet Uz Training and Research Hospital, Department of Pediatric Endocrinology, Izmir, Turkey; 12 Ankara City Hospital, Department of Pediatric Endocrinology, Ankara, Turkey; 13 College of Health & Life Sciences, Hamad Bin Khalifa University, Qatar Foundation, Doha, Qatar; 14 Department of Pediatrics, Division of Endocrinology, Sidra Medicine, Doha, Qatar; 15 Maramara University Faculty of Medicine, Department of Pediatric Endocrinology, Istanbul, Turkey

**Keywords:** *PTF1A* gene, permanent, neonatal diabetes, pancreas agenesis/hypoplasia, cholestasis

## Abstract

**Context:**

Biallelic mutations in the *PTF1A* enhancer are the commonest cause of isolated pancreatic agenesis. These patients do not have severe neurological features associated with loss-of-function *PTF1A* mutations. Their clinical phenotype and disease progression have not been well characterized.

**Objective:**

To evaluate phenotype and genotype characteristics and long-term follow-up of patients with *PTF1A* enhancer mutations.

**Setting:**

Twelve tertiary pediatric endocrine referral centers.

**Patients:**

Thirty patients with diabetes caused by *PTF1A* enhancer mutations. Median follow-up duration was 4 years.

**Main Outcome Measures:**

Presenting and follow-up clinical (birthweight, gestational age, symptoms, auxology) and biochemical (pancreatic endocrine and exocrine functions, liver function, glycated hemoglobin) characteristics, pancreas imaging, and genetic analysis.

**Results:**

Five different homozygous mutations affecting conserved nucleotides in the *PTF1A* distal enhancer were identified. The commonest was the Chr10:g.23508437A>G mutation (n = 18). Two patients were homozygous for the novel Chr10:g.23508336A>G mutation. Birthweight was often low (median SDS = –3.4). The majority of patients presented with diabetes soon after birth (median age of diagnosis: 5 days). Only 2/30 presented after 6 months of age. All patients had exocrine pancreatic insufficiency. Five had developmental delay (4 mild) on long-term follow-up. Previously undescribed common features in our cohort were transiently elevated ferritin level (n = 12/12 tested), anemia (19/25), and cholestasis (14/24). Postnatal growth was impaired (median height SDS: –2.35, median BMI SDS: –0.52 SDS) with 20/29 (69%) cases having growth retardation.

**Conclusion:**

We report the largest series of patients with diabetes caused by *PTF1A* enhancer mutations. Our results expand the disease phenotype, identifying recurrent extrapancreatic features which likely reflect long-term intestinal malabsorption.

Pancreas transcription factor-1-alpha, encoded by the *PTF1A* gene, is a basic helix–loop–helix (bHLH) protein essential for the development of the pancreas and cerebellum (1, 2). Homozygous loss-of-function mutations in *PTF1A* have been reported in 4 cases with a severe phenotype of pancreatic agenesis, resulting in permanent neonatal diabetes (PNDM) and pancreatic exocrine dysfunction, and a severe neurological phenotype with developmental delay, central hypoventilation, and complete cerebellar agenesis associated with a survival period of up to 4 months (3-5). A similar phenotype was identified in a *Ptf1a*^–/–^ mouse model, where the severity of pancreatic hypoplasia, pancreatic exocrine insufficiency, and glucose intolerance was shown to correlate with the *Ptf1a* mRNA levels, suggesting a dosage-dependent effect (1).

Isolated pancreatic agenesis/hypoplasia can also result from biallelic mutations in the gene encoding the transcription factor PDX1 (pancreatic and duodenal homeobox 1). These patients present with PNDM and exocrine insufficiency, which ranges in severity from an overt presentation requiring full pancreatic enzyme replacement to subclinical disease. A possible genotype–phenotype correlation has been suggested to explain this variability (6-10). Marked phenotypic variability has also been reported in patients with mutations in other pancreatic agenesis genes, such as *GATA6, GATA4*, and *HNF1B*, which encode for fundamental pancreatic transcription factors (11-14).

In 2014, Weedon et al. combined linkage analysis, genome sequencing, and epigenomic annotation to identify 6 novel mutations in a previously unknown ~400 bp enhancer, 25 kb downstream of the *PTF1A* gene (15). All 14 patients with biallelic mutations in this pancreas-specific *PTF1A* enhancer had isolated pancreatic agenesis//hypoplasia resulting in PNDM and exocrine insufficiency requiring pancreatic enzymes supplementation (15-18).

In this study, we assess the presenting clinical characteristics, genotype–phenotype relationships, long-term glycemic outcome, and growth status of the largest patient series with *PTF1A* distal enhancer mutations described to date.

## Patients and Methods

### Patient cohort

Thirty patients homozygous for mutations in the *PTF1A* distal enhancer referred to 12 pediatric endocrine centers were included in the study. Information regarding the clinical presentation, birth and family history, phenotype, biochemical data, pancreatic imaging, and treatment (the type of insulin, doses, and mode of administration) were retrospectively collected using a standardized proforma. Follow-up data reporting most recent clinical characteristics, growth status, treatment, glycemic outcome, and neurodevelopmental milestones were also reviewed.

### Clinical evaluation

Delivery before 36 weeks of gestation was defined as premature birth while those after 36 weeks were classified as term. Patients born with a weight standard deviation score (SDS) below –2 were considered to have intrauterine growth restriction (IUGR) (19). Growth status during follow-up was assessed by measurement of latest height/length and calculation of age–sex-adjusted SDS using population standard references (20). A height SDS below –2 was considered as short stature. For patients over 2 years of age, body mass index (BMI) and age–sex-adjusted BMI z-score were calculated using the standard formula and population standard references. A BMI z-score below –2 was considered underweight, BMI z-score between –2 and +2 was considered as normal, and BMI z-score >+2 was considered obese.

The pancreatic exocrine function was evaluated based on clinical features of pancreatic insufficiency such as having steatorrhea, intestinal malabsorption, and failure to thrive. For biochemical confirmation, when applicable, fecal elastase and pancreatic enzymes (pancreatic amylase and lipase) were measured and assessed according to laboratory-specific reference values. Exocrine pancreas insufficiency was treated with pancreatic enzyme replacement.

The diagnosis of cholestasis was considered based on clinical (acholic stool) and biochemical features, including elevated conjugated bilirubin, gamma-glutamyl transferase (GGT) and alkaline phosphatase (ALP). Cases with cholestasis were treated with ursodeoxycholic acid (UDC) when indicated. Liver transaminases (alanine aminotransferase, ALT, and aspartate aminotransferase, AST) were classified according to the upper limit of normal (ULN) for the laboratory. To account for interlaboratory variability and avoid overestimation, we assigned the lower limit as 50 IU for all patients. Values from 50 IU to 5 × ULN were classified as mild, from 5 × ULN to 10 × ULN as moderate and values above 10 × ULN were considered as severely elevated transaminases (21).

The glycemic outcome was assessed based on the latest glycated hemoglobin (HbA1c). In keeping with the International Society of Paediatric and Adolescents Diabetes guidelines (22), we categorized glycemic status based on whether patients had achieved an HbA1c below 7.0% (22).

The pancreatic size was assessed by imaging using abdominal ultrasonography, computed tomography, or magnetic resonance imaging in all patients. Cranial imaging and neurodevelopmental status of patients were reported by the clinicians but were not specifically tested for in the present study.

### Genetic analysis

Molecular genetic analysis was performed in all cases. Briefly, genomic DNA was extracted from peripheral leukocytes of patients using standard procedures. All 28 known neonatal diabetes genes and the *PTF1A* distal enhancer were analyzed using a custom-designed targeted next-generation sequencing assay and sequenced on an Illumina HiSeq2000 or NextGene 500. Details of the methodology have been reported previously (23). Testing of parental samples was performed by Sanger sequencing (primers available on request). The genomic location of the enhancer variants is reported according to the GRCh37 Genome Reference Consortium Human Build 37 (hg19).

### Ethics

The study was performed with the ethics approval of the institutional review board of Ataturk University Medical Faculty (document number: 30.05.2019/101) and in accordance with the principles of the Declaration of Helsinki with a written informed consent given by the patients or their legal guardians.

### Statistical analysis

Statistical analysis was performed using IBM SPSS 22.0 for Windows statistical software. Shapiro–Wilk test was used to test the normality distribution of the data. Ratios were compared using the chi-squared (or Fisher exact) test. Means were compared using the independent sample t-test in normally distributed data, and medians were compared using the Mann–Whitney U test for non-normally distributed data. The Wilcoxon signed-rank test was performed for comparison of repeated measures. Spearman’s rank correlation analysis was performed. Data were expressed as number (%) and median (first; third quartiles). A *P*-value ≤.05 was considered as statistically significant.

## Results

We recruited 30 patients (19 male) from 25 families who were homozygous for a mutation in the *PTF1A* distal enhancer. Of those, 6 patients have been previously described (18, 24). Consanguinity was reported in 23 families (92%). Nine of 29 (31%) patients were born premature (<36 weeks’ gestation). The median duration of follow-up was 4.08 (2.58; 8.37) years.

The presenting clinical characteristics and follow-up data for the cohort are summarized in Table 1 and (25). All supplementary material and figures are located in a digital research materials repository (25).

**Table 1.** Presenting and follow-up characteristics of cases with *PTF1A* distal enhancer mutations

	Median (IQR1-IQR3)
**Birth**	
Gestational age (week) (n = 29)	36 (32; 38)
Birth weight (g) (n = 29)	1510 (1345; 1815)
Birth weight (SDS)(n = 29)	–3.4 (–5.1; –2.3)
**Diabetes**	
Age at diagnosis (days) (n = 30)	5 (1.8; 20.2)
Blood glucose at presentation (mmo/L) (n = 29)	22.8 (15.5; 28.8)
C-peptide (pmol/L) (n = 26)	33.3 (3.33; 33.3)
Initial insulin dose (U/kg/day)(n = 27)	1 (0.8; 1.0)
Current insulin dose (U/kg/day) (n = 25)	0.8 (0.6; 0.9)
Latest HbA1c (n = 28)	9.3 (8.5; 10.0)
**Other biochemistry**	
Hemoglobin (gr/dL)(n = 25)	8.2 (7.6; 11.8)
Ferritin (mg/dL)(n = 12)	1415 (869; 1696)
**Follow-up data current age (months) (n = 30)**	61 (30.5; 103)
Duration of follow-up (months) (n = 29)	49 (31; 100.5)
Latest height/length (SDS) (n = 29)	–2.35 (–3.22; –1.54)
Latest BMI (kg/m2) (n = 25)	15.3 (14.6; 16.7)
Latest BMI z-score (n = 25)	–0.52 (–1.18; 0.38)

### Molecular genetics

**Figure 1.**
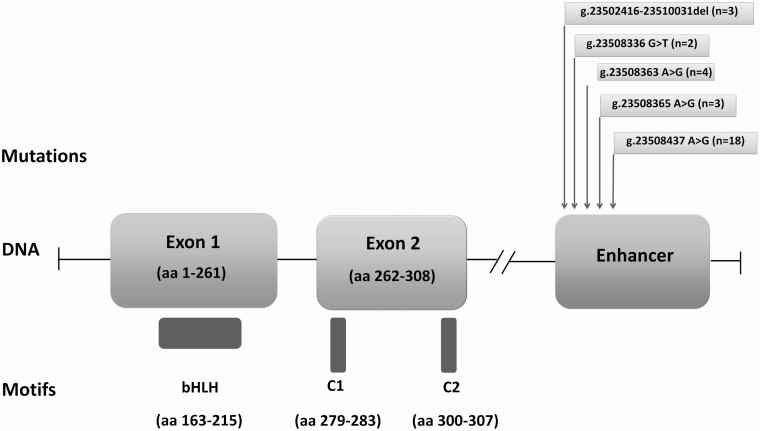
Graphical representation of the PTF1A protein motifs and of the *PTF1A* gene. The pancreatic progenitor-specific enhancer region is located approximately 25 kb downstream of the coding sequence of *PTF1A*. The positions of the mutations detected in our cohort are displayed (number in parentheses indicates the number of cases with each mutation detected in our cohort).

The most common mutation was the previously reported *PTF1A* enhancer Chr10:g.23508437A>G variant, which was detected in 18 cases (60%) (Fig. 1). Three previously reported distal enhancer mutations were detected in 10 individuals, and a novel mutation affecting the *PTF1A* enhancer (Chr10:g.23508336G>T) was identified in 2 patients (Table 2 and Fig. 1).

**Table 2.** Pancreatic and extrapancreatic features of 30 cases with diabetes due to a *PTF1A* enhancer mutation

Mutation	g0.23508437A>G	g0.23508363A>G	g0.23508336G>T^*a*^	g0.23502416-23510031del	g0.23508365A>G	Total n (%)
Number of cases	18	4	2	3	3	30 (100)
PNDM/DM^*b*^ (n = 30)	18	4	2	3	3	30 (100)
Pancreas agenesis/hypoplasia (n = 30)	18 (100%)	4 (100%)	2 (100%)	3 (100%)	3 (100%)	30 (100%)
Exocrine pancreas insufficiency (clinical) (n = 30)	18 (100%)	4 (100%)	2 (100%)	3 (100%)	3 (100%)	30 (100%)
Exocrine pancreas insufficiency (biochemical) (n = 19)	12 (100%)	3 (100%)	1 (100%)	1 (100%)	2 (100%)	19 (100%)
Cholestasis (n = 24)	9 (60%)	2 (50%)	1 (50%)	NA	2 (66.7%)	14 (58.3%)
Elevated transaminases (n = 25)	11 (68.8%)	3 (75%)	1 (50%)	NA	3 (100%)	18 (72%)
Anemia (n = 25)	11 (68.8%)	4 (100%)	2 (100%)	NA	2 (66.7%)	19 (76%)
Hyperferritinemia (n = 12)	7 (100%)	4 (100%)	1 (100%)	NA	NA	12 (100%)
Growth retardation at follow-up (n = 29)	12 (70.6)	3 (75%)	1 (50%)	2 (66.7%)	2 (66.7%)	20 (69%)
HbA1c >7% (n = 28)	16 (94.1%)	3 (75%)	2 (100%)	3 (100%)	2 (100%)	26 (92.9%)

n = in first column indicates the number of patients assessed for each feature.

Abbreviation: NA, data not available.

^*a*^Novel mutation.

^*b*^Diabetes was detected in the neonatal period (PNDM) in 28 patients and later in life in 2 patients (19 months and 11 years old in 2 cases with distal enhancer Chr10:g.23508437A>G *PTF1A* mutation).

### Fetal growth

Fetal growth was markedly reduced [median birth weight SDS –3.42 (–5.08; –2.30)] with 25/29 (86.2%) cases having IUGR. This is consistent with severe insulin deficiency in utero.

### Postnatal growth

Longitudinal growth postnatally was markedly reduced with the median height/length SDS measured at the latest follow-up visit being –2.4 (–3.2; –1.5); height SDS was equal or below –2 in 20 of 29 (69%) cases. Reduced height occurred early, with a similar median length SDS of cases below the age of 2 years [n = 4; –3.0 (–4.91; –1.65)] and height SDS of cases ≥2 years old [n = 25; –2.2 (–3.16; –1.63)] (*P* = .487). No correlation was observed between the latest height SDS versus birthweight SDS (ρ = –0.161; *P* = .412) and latest height SDS versus current age (ρ = 0.301; *P* = .113).

Weight was reduced in proportion to height: the median BMI z-score for the 25 patients who were ≥2 years old at the last follow-up visit was –0.52 (–1.18; 0.38). In just 1/25 patient, the BMI z-score was above +2 and in 1/25 below –2.

### Diabetes features

The median age at diagnosis of diabetes was 5 days (1.75; 20.25), with 28 patients diagnosed with PNDM before the age of 6 months. Two unrelated patients were diagnosed with diabetes outside the neonatal period, at the ages of 19 months and 11 years. Both patients were reported to have shown symptoms of exocrine insufficiency since infancy which was biochemically confirmed at diabetes diagnosis. Both are homozygous for the Chr10:g.23508437A>G enhancer mutation. Diabetes autoantibodies were negative in all patients. C-peptide was measured in 26 patients, and it was <200 pmol/L in all of them.

Parental consanguinity was reported in 23/25 (92%) families. Seven of the 25 (28%) families had a history of PNDM/monogenic diabetes. In 6 families there were siblings either with confirmed neonatal diabetes or perinatal elevated blood glucose who had died due to unknown causes, suggesting a missed diagnosis of NDM presumably due to the same *PTF1A* mutations (DNA samples were not available for testing).

### Management strategies and glycemic outcome

All patients were treated with replacement doses of insulin (median initial dose 1 U/kg/day). In the neonatal period, multidose regular insulin or NPH (neutral protamine hagedorn) insulin injections were initially administered. In 6 patients, the insulin infusion pump was successfully used. All remaining 24 patients were treated with basal bolus insulin at follow-up. Patients insulin requirement was decreased with median initial versus latest insulin dose of 1.0 (0.8; 1.0) versus 0.8 (0.6; 0.9) respectively (*P* = .009). The median HbA1c was 9.3% (8.5; 9.97) with only 2/28 (7.1%) patients having achieved an HbA1c level below 7.0%.

### Pancreas imaging and exocrine function

Pancreas imaging confirmed pancreatic agenesis/hypoplasia in all 30 patients. All patients had clinical/subclinical signs of exocrine pancreas insufficiency, including steatorrhea and failure to thrive. This was biochemically confirmed in 19 patients by low fecal elastase, pancreatic enzymes (lipase, pancreatic amylase), hypoalbuminemia, and low fat-soluble vitamins (A, D, E) (Table 1). All patients were treated with pancreatic enzymes.

### Neurodevelopmental outcome

Although neuroimaging and neurodevelopmental status were not specifically evaluated in each patient, a review of hospital files identified neurological features in 7 patients. Mild neurodevelopmental delay was present in 4 patients, of which 2 also had epilepsy, 1 had congenital cataract, and 1 had developmental delay only. Two patients had epilepsy alone. One individual had microcephaly, global developmental delay, and epilepsy (25). Cranial imaging was available in 16 patients. No patients had cerebellar aplasia: 14/16 patients had normal cranial imaging, 1 patient had ventricular dilatation, and 1 had hypoplasia of corpus callosum and periventricular leukomalacia (25).

### Extrapancreatic features

A transient, but markedly elevated ferritin level was detected in 12/12 patients for whom a measurement was available at diagnosis during the neonatal period. All patients with elevated ferritin also had anemia which required blood transfusion in 9/12 (75%). In total, anemia was detected in 19 of 25 (76%) patients for whom a complete blood count was available at diagnosis. Of those, 1 patient who was diagnosed at the age of 19 months had hypochromic microcytic anemia due to iron deficiency. All patients who were diagnosed in the neonatal period and had anemia detected beyond the physiological anemia period had normocytic anemia. The etiological evaluation did not detect a specific reason (including hemolysis or micronutrient deficiency). In total, 12 patients required multiple blood transfusions due to severe anemia (25).

A transient mild to severe elevation in liver transaminases, which recovered spontaneously after replacement of UDC or pancreatic enzymes, was detected in 18 of 25 (72%) patients. Clinical and biochemical features of cholestasis (conjugated hyperbilirubinemia, GGT, ALP, and acholic stool) was detected in 14/24 (58.3%) patients. Of those, cholestasis spontaneously resolved in 3 patients after replacement of pancreatic enzymes (Creon^®^), while 11 patients required UDC treatment. In 1 patient with cholestasis, there was an extremely elevated creatinine kinase (>42.000 IU) which spontaneously resolved after replacement of pancreatic enzymes.

### Genotype–phenotype relationship

There was phenotypic variability in our cohort, with 2 patients diagnosed with diabetes after the age of 6 months and 25 patients presenting additional extrapancreatic features. This variability was often observed between individuals with the same mutation and, in some cases, within the same family. In fact, cases 4.2 and 22.2 in our cohort, who were diagnosed at the age of 19 months and 11 years respectively, both had siblings homozygous for the same Chr10:g.23508437A>G mutation who were diagnosed with PNDM and pancreatic agenesis. In contrast, individuals 4.2 and 22.2 not only developed diabetes later but had subclinical exocrine pancreas insufficiency and did not have short stature (height SDS + 0.15 and +0.65 respectively). These data do not support a direct genotype–phenotype relationship, but investigation of additional families is needed to define whether additional factors influence the phenotypic presentation.

The 2 patients homozygous for the novel Chr10:g.23508336A>G mutation had a similar phenotype to the other patients with previously reported *PTF1A* enhancer mutations, supporting this mutation’s causality.

## Discussion

We have evaluated the presenting clinical characteristics, molecular genetics, and long-term follow-up of the largest series to date of patients with diabetes and pancreatic agenesis due to *PTF1A* distal enhancer mutations. We show that neurological features are not a major characteristic of these patients and describe novel clinical findings of anemia, hyperferritinemia, and cholestasis as prominent early features, thereby expanding the spectrum of extrapancreatic features associated with *PTF1A* distal enhancer mutations.

Homozygous truncating mutations in *PTF1A* cause a severe syndrome of PNDM, central hypoventilation, pancreatic agenesis, exocrine pancreas dysfunction, and complete cerebellar agenesis associated with a very short survival period (3-5). Only 4 cases with homozygous loss-of-function *PTF1A* mutations have been reported so far.

In 2014, Weedon et al. identified biallelic mutations in a noncoding ~400 bp genomic region located 25 kb downstream of *PTF1A* in 14 patients (15). All patients had isolated pancreatic agenesis without cerebellar involvement or other extrapancreatic features. This phenotype and epigenetic data suggested that this enhancer is pancreas specific and functional analysis demonstrated that the base substitutions disrupted enhancer activity by abolishing transcription factor (FOXA2 and PDX1) binding (15). After the initial report, 8 further cases have been published (16-18, 26, 27). Overall these cases appear to be more common than those with biallelic truncating *PTF1A* variants, likely due to the higher survival rate resulting from the lack of severe neurological features. The most prevalent mutation in our series was the chr10:23508437A>G, which has previously been reported in cases from Turkey (15) suggesting a possible founder effect. In our study, we identified a novel Chr10:g.23508336A>G mutation within the *PTF1A* distal enhancer in 2 patients with PNDM and pancreas agenesis, further emphasizing the importance of this regulatory region in pancreatic development.

Our study has expanded the phenotype associated with *PTF1A* distal enhancer mutations. In the initial report, only 1/14 patients developed fatal intrahepatic cholestasis and liver failure. In our series, intrahepatic cholestasis was detected in 58.3% of patients (Table 2). In all patients, cholestasis resolved with the introduction of pancreas enzyme and/or UDC. Regardless of having or not having cholestasis, a transient mild to severely elevated transaminases were present in 72% (18/25) of patients suggesting a hepatocellular injury.

Another common feature was anemia which was detected in 76% of cases (n = 19). Of these, 12 had severe anemia requiring multiple blood transfusions of unknown cause. Furthermore, we report the detection of elevated ferritin level in 12 patients. In the first patient (Patient 16.1) in whom we observed high ferritin level, a diagnosis of neonatal hemochromatosis was suspected but excluded by cardiac and abdominal magnetic resonance imaging and liver biopsy. The ferritin level spontaneously decreased to within the normal range during follow-up. Elevated and spontaneous normalization of ferritin level in intrahepatic and extrahepatic cholestasis has been suggested as a result of decreased hepatic uptake or clearance for ferritin (28). However, some of our patients with hyperferritinemia did not have cholestasis. Furthermore, all patients with hyperferritinemia also had anemia that required blood transfusion in the vast majority (9/12). While elevated ferritin has been suggested to suppress hematopoietic progenitor cells, there is currently insufficient evidence to explain the clinical relevance of elevated ferritin and anemia in our cases (29). Elevated ferritin level was detected in all cases for whom a ferritin measurement was available at diagnosis during the neonatal period, suggesting that measurement of ferritin level could potentially be a diagnostic tool in highlighting patients with PNDM who develop clinical or subclinical cholestasis, hepatocellular dysfunction, or exocrine pancreas insufficiency. While ferritin is also a marker of inflammation, there was no evidence of infection or inflammation in our cases with hyperferritinemia. Further studies are needed to understand the biological mechanism behind this novel finding as well as its validity and impact on patients’ diagnosis.

The majority of patients with *PTF1A* distal enhancer mutations presented in the early neonatal period with IUGR suggesting in utero insulin deficiency (15-18, 26, 27, 30). Two of our cases presented after the neonatal period, at the ages of 19 months and 11 years. This is in keeping with previously published cases with the Chr10:g.23508437A>G *PTF1A* enhancer mutation, for whom intrafamilial variability was observed with some individuals developing diabetes outside the neonatal period and having subclinical exocrine pancreas insufficiency (15, 17). Since all the previously reported cases with a delayed diagnosis of diabetes had the Chr10:g.23508437A>G *PTF1A* distal enhancer mutation, it is possible that this variant does not entirely abolish the enhancer activity and causes pancreas hypoplasia with some residual beta-cell mass. However, this is also the most common enhancer variant identified to date, and there are currently insufficient data to evaluate a possible genotype–phenotype correlation among the different enhancer mutations. Therefore, the mechanism by which these cases had a milder phenotype than their siblings and other individuals with identical *PTF1A* distal enhancer mutations is yet to be elucidated.

**Figure 2.**
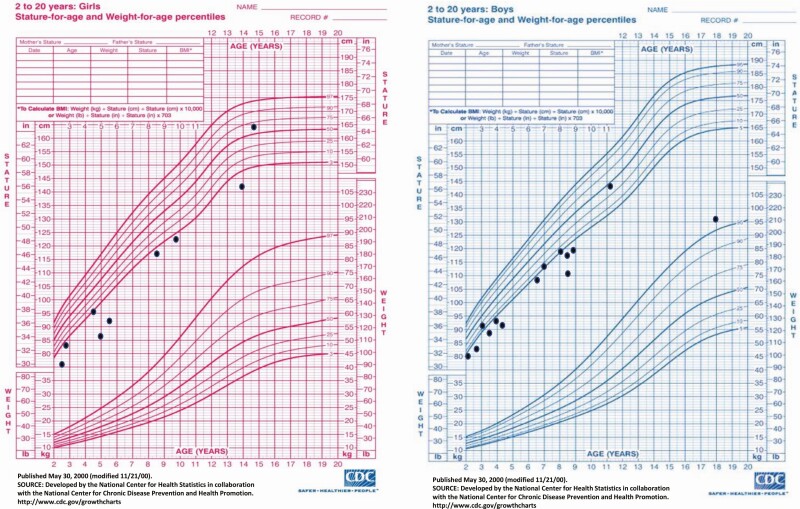
Sex-specific growth charts displaying the latest height of 25 cases over 2 years old, indicating that the majority of cases failed catch-up growth and have short stature.

Data on the long-term growth outcome in patients with distal enhancer *PTF1A* mutations have so far been scarce. In some case reports of patients with enhancer mutations, catch-up growth in the infancy period has been reported (16, 17, 26, 27). Nevertheless, the majority of these reports did not provide a long-term follow-up. In our series, we evaluated the growth status for a median duration of 4 years (oldest patient now 18 years old) and found that the majority of cases have failure to thrive and short stature despite treatment with replacement doses of insulin and pancreatic enzymes (Fig. 2). There was no correlation between height SDS and the latest age when patients were assessed, suggesting no improvement in growth with age. Different factors may have affected growth in the patients in our cohort. These include poor glycemic control, as 26/28 patients in our cohort failed to achieve HbA1c <7%. Mean HbA1c >7% in individuals with type 1 diabetes has been previously suggested to result in severely reduced adult height (31). Furthermore, our patients’ growth could have been affected by inadequate pancreatic enzyme replacement, which is known to be very difficult in children with pancreatic agenesis and a chronic disease such as diabetes which can negatively influence growth. Besides, since some cases had subclinical exocrine pancreas insufficiency, this diagnosis might have been missed until confirmation of the genetic etiology. This strongly supports the importance of genetic analysis for prompt diagnosis and appropriate management of cases with pancreatic agenesis and diabetes. Finally, to exclude the potential negative impact of intestinal malabsorption due to exocrine pancreas insufficiency, we assessed the latest BMI z-score, which revealed that only one case was underweight. This suggests that the effect on linear growth is unlikely to be due to intestinal malabsorption and inadequate calorie intake. Therefore, there might be some other growth-promoting factors deficiencies or regulatory gene networks affecting growth in these patients. Additional studies investigating data on linear growth as well as a growth hormone stimulation test will be needed to rule out growth hormone deficiency in patients with short stature.

Although we did not specifically investigate neurological features in our cohort, some minor changes in cranial imaging, epilepsy, and mild to moderate neurodevelopmental delay were identified in 8 cases. However, imaging did not detect abnormalities in the cerebellar region in any of our patients. As these features were not consistently present in the cohort, it is unlikely that they are directly caused by the *PTF1A* enhancer mutations. It is, however, worth noting that the majority of our patients had various clinical issues (born premature, IUGR, exocrine pancreas insufficiency, poorly controlled diabetes and probably high degree of glycemic variability from early infancy) which may have contributed to their neurodevelopmental delay.

In conclusion, our results expand the clinical phenotype associated with *PTF1A* enhancer mutations. In this large series of patients with *PTF1A* distal enhancer mutations, we highlight the importance of genetic testing and clinical follow-up studies to characterize rare genetic subtypes of diabetes.

## Data Availability

PTF1A mutation details have been deposited in the Decipher database (https://decipher.sanger.ac.uk/). The datasets generated during and/or analyzed during the current study are not publicly available but are available from the corresponding author on reasonable request.

## Abbreviations

ALP: alkaline phosphatase
ALT: alanine aminotransferase
AST: aspartate aminotransferase
bHLH: basic helix–loop–helix
BMI: body mass index
GGT: gamma-glutamyl transferase
HbA1c: glycated hemoglobin
IUGR: intrauterine growth restriction
PNDM: permanent neonatal diabetes
SDS: standard deviation score
UDC: ursodeoxycholic acid
ULN: upper limit of normal

## Additional Information

***Disclosure Summary:*** The authors have no any conflict of interest to declare.
